# Measuring sport experiences in children and youth to better understand the impact of sport on health and positive youth development: designing a brief measure for population health surveys

**DOI:** 10.1186/s12889-018-5325-9

**Published:** 2018-04-03

**Authors:** John Cairney, Heather J. Clark, Matthew Y.W. Kwan, Mark Bruner, Katherine Tamminen

**Affiliations:** 1 Faculty of Kinesiology and Physical Education, University of Toronto, 55 Harbord St, Toronto, ON M5S 2W6 Canada; 2 Department of Family Medicine, McMaster University, 100 Main Street West, Hamilton, ON L8P 1H6 Canada; 3 School of Physical and Health Education, Nipissing University, 100 College Drive, Box 5002, North Bay, ON P1B 8L7 Canada

**Keywords:** Youth sport participation, Youth experiences in sport, Measurement of sport, Quality sport

## Abstract

**Background:**

Despite the proliferation of studies examining youth sport participation, there are significant gaps in knowledge regarding the impact of youth sport participation on health and development. These gaps are not new, but have persisted due to limitations with how sport participation is measured. Much of the research to date has measured sport participation as binary (yes/no) or count measures. This has been especially true in survey-based research. Yet, at the same time, research has investigated youths’ experiences in sport such as the influence of coaches, teammates, and parents. The ability to measure these experiences is constrained by the need to use a number of measures along with gaps in the content covered in existing measures. We propose to develop and test the *Sport Experiences Measure: Children and Youth* (SEM:CY) as a population survey-based measure that captures the salient aspects of youths’ experience in sport.

**Methods:**

The SEM:CY will be developed and tested across three phases. Phase I includes qualitative research with members of the sport community and engagement with an expert group to generate and obtain feedback on the initial item pool. In Phase II will recruit two consecutive samples of students from schools to complete the draft measure. Analysis will focus on assessing the items and factor structure of the measure. Factor structure will be assessed first with exploratory factor analysis and then confirmatory factor analysis. In phase III we will test the association between the SEM:CY with a measure of perceived competence, sport anxiety, and positive youth development to assess construct validity. We will also examine whether the structure of the measure varies by age or gender.

**Discussion:**

The SEM:CY measure will provide a meaningful contribution to the measurement and understanding of youth sport participation. The SEM:CY can be used as a stand-alone measure to understand youth experiences in sport programs, or in combination with other health and development measures to better understand how youth sport can contribute to both positive and negative outcomes.

## Background

From a public health perspective, sport participation is one important form of physical activity for children and youth. In 2008 for example, 71% of Canadian children ages 6-9 and 84% of children ages 10-13 reported participating in organized sport over the course of 1 year [1]. Similar rates were observed for American children and youth, with 69% of girls and 75% of boys reporting participation in organized sport [2]. Beyond participation in physical activity, organized sport (i.e., participation in a sport with a coach) has been continually identified as an important context that can support the health, development, and well-being of youth [3–5].

Despite this potential, researchers have reported inconsistent and sometimes contradictory findings concerning the health and developmental impacts of sport participation. For example, recent systematic reviews have shown that while sport participation may be associated with lower use of illicit drugs, rates of alcohol use among youth athletes is higher than among non-athlete peers [6, 7]. Mixed results have also been reported in reviews focusing on other outcomes, such as self-esteem, sense of competence, and delinquency: some studies show a positive relationship, other studies show no association or even negative impacts of participation [8–11].

While there are many potential explanations for these discrepant findings, authors have continually pointed to issues concerning the measurement of sport participation [5, 7–9, 12]. The sport literature has been dominated by cross-sectional research that has measured sport participation as a binary (yes or no) or count (number of sports played) variable and examined relationships with developmental and health outcomes. This has been especially true of survey-based research in sport [7–9]. Such blunt measures conceal tremendous variability in experiences with sport, leaving the impact of sport on health and development as essentially a black box [12].

Examples from qualitative research illustrate just how much experiential information is lost when sport is measured this way. Camiré, Trudel, and Forneis, for example, reported young athletes felt sport provided them with opportunities for leadership and life skill development [13]. In contrast, Buford-May documented incidents of racism in youth sport participation [14]. We would expect different health and developmental outcomes associated with experiences of leadership and life skills versus racism, but current survey measures would classify youth in both studies as athletes (i.e., “yes” to a question on sport participation) and therefore fail to capture important variability in their experiences.

To address this gap in the current literature, we propose to create a *Sport Experiences Measure for Children and Youth* (SEM:CY) to capture salient experiences in sport that may better predict positive and negative developmental outcomes among youth. Furthermore, we propose to design the measure specifically for survey-based population health research. This way, the measure could be added to existing surveys that monitor youth health and development. For example, such a measure could be added to on-going surveillance and longitudinal adolescent health surveys such as The National Longitudinal Study of Adolescent to Adult Health [15], Adolescent Health Survey [16], Canadian Tobacco Use Survey [17], and the Health Behavior of School Aged Children survey [18]. Adding the SEM:CY to existing surveys would greatly increase the availability and quality of data on youths’ experiences in sport to help advance research beyond mixed results to a more refined understanding of when and how sport participation impacts youth development.

To be useful in survey-based health research, the measure must be self-reported, comprehensive, and brief. The measure needs to be self-reported as this is the most common method used in population health surveys and is also well-suited to capture subjective experiences. A comprehensive measure is required to fully capture the multiple facets of youths’ experience in sport. The measure must be brief to be included in existing and future population health surveys, which include a number of other measures and have limited space for additional questions [19]. Currently, no measure meeting these criteria exists.

### Understanding youths’ experiences in sport

The impact of youth sport has garnered significant interest across disciplines, with studies examining the potential health impacts of sport participation [20, 21], academic impacts [22, 23], social and psychological impacts on the youth [7], impacts on delinquency [10], and societal benefits [24, 25]. Measuring youths’ experience in sport is essential to advance research across disciplines. To develop the SEM:CY, we will draw heavily from the positive youth development (PYD) in sports literature. Youth experience in sport has garnered significant attention in the PYD in sport literature. PYD is an area of psychology that is interested in the competencies and processes that result in positive development [3].

**Fig. 1:**
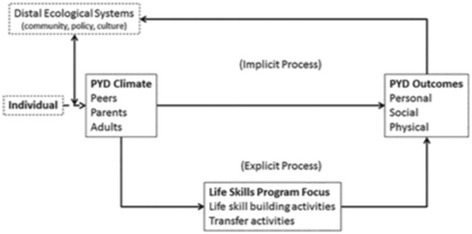
Holt and colleagues Model of Positive Youth Development through Sport [27]

Recently, Holt and colleagues’ [26] developed the *Grounded Theory of Positive Youth Development through Sport* model based on a recent synthesis of 63 qualitative studies on positive youth development through sport. This model includes distal social-ecological systems and individual characteristics that influence the sport, features of the sport climate that impact sport experiences, and program focus that contribute to positive youth development outcomes (see Fig. 1).

Consistent with our focus on youths’ subjective experience in sport (more proximal influences), we focus on the three elements of the sport climate that support positive youth development outcomes in the model: relationships with coaches (identified as adults in the model), relationships with teammates (identified as peers in the model), and involvement of parents. As our intention for the SEM:CY is to apply to outcomes beyond PYD, we also draw on the broader sport literature to ensure comprehensive coverage of youth sport experiences. Not surprisingly, the importance of relationships with coaches, parents, and teammates in sport has also been identified in the broader sport literature as essential to youths’ experiences in sport [5, 27–31]. Additionally, other influencers such as family members other than a parent (e.g., siblings), parents of others on the team, and spectators at games has been identified as important to youth’s sport experience, though they have received less attention in the literature to date [9, 32].

Most research on youths’ sport experience has focused on the role of the coach. In particular, the importance of the coach in establishing the motivational climate of the sport is one of the most established aspects of youths sport experience. Motivational climate is grounded in *Achievement Goal Theory* [33, 34] and considers different ways for interpreting competence: self- referenced competence that focuses on skill development and mastery and external referenced competence that evaluated competence in reference to others (e.g., winning and comparing teammates to each other). When coaches that emphasize skill development and mastery, youth are more likely to show positive outcomes whereas coaching behaviours that emphasize winning and comparisons with others are associated with negative outcomes including anxiety and stress, conflict with peers, less sportsman-like behaviour, and negative developmental outcomes [35].

Teammate and parents are also important in establishing the motivational climate of the sport. While the role of both teammates and parents have received less empirical attention than coaches, [35] the impact of both teammates and parents has been demonstrated independent of the influence of coaches [36–39]. When teammates emphasis skill development and mastery (i.e., self-referenced motivational climate), youth report more enjoyment of sport, pro-social sport attitudes (e.g., congratulating an opponent after a loss) and fewer antisocial sport attitudes (e.g., attitudes towards cheating and gamesmanship). The same pattern of results has been found with parental motivational climate: emphasis of skills is related to positive outcomes, while emphasizing winning is related to negative outcomes [35].

The importance of the coach-athlete relationship is another established element of youths’ sport experience. A positive relationship and rapport between athletes and coaches is related to a number of positive developmental outcomes including higher perceived competence, pro-social norms, and lower stress whereas negative rapport with coaches is associated with higher stress and negative peer dynamics [40–42]. There is a dearth of research on the relationship of teammates and parents relative to coaches. However, the research that has been conducted reports positive relationships with teammates and parents in sport is associated with positive development including perceived physical competence and personal and social skills [43–46].

While there is recognition that coaches, teammates, and parents each play an important role in youths’ sport experience, there is some evidence of other influences on youth sport experience. For example, positive spectator behaviours (e.g., cheering for members of both teams) and negative spectator behaviours (e.g., taunting players or arguing with officials) has been associated with the sportsmanlike behaviour of young athletes during games [32]. We propose that these types of behaviours can influence the motivational climate of the sport. Arguing or aggressiveness towards officials can indicate that winning is highly valued whereas recognizing skill and achievements on both teams can indicate the value of skill. Similar to relationships within sport, the impact of relationships with other family members (e.g., siblings) and peers aside from teammates is an area that has been identified as warranting further investigation [11].

In summary, a sport experience where coaches, teammates, parents, and others engage in behaviours that foster skill development, do not overemphasize winning, and youth have positive relationships is likely to produce a more quality sport experience and enhance developmental outcomes. Alternatively, an experience that focuses on winning, where negative behaviour is modeled, and there are conflicts with relationships is more likely to produce a lower quality experience and be associated with negative developmental outcomes.

**Fig. 2:**
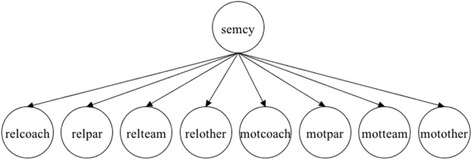
Initial conceptual model for Sport Experiences Measure for Children and Youth. relcoach: relationships with coaches, relpar: relationships with parents, relteam: relationships with teammates, relother: relationships with others, motcoach: coach motivational climate, motpar: parental motivational climate, motteam: teammate motivational climate, motother: other motivational climate

Based on this review, we propose an initial conceptual model for the SEM:CY measure that includes the following domains: relationships with coaches, relationships with parents, relationships with teammates, relationships with others, coach motivational climate, parental motivational climate, teammate motivational climate, and other motivational climate (see Fig. 2). This model will act as a starting point for item generation and will be thoroughly tested and refined through expert input and statistical analysis as detailed in our methodology.

### SEM:CY in relation to existing measures

While there are a number of surveys that capture some elements of youths’ experience of sport, there is no single measure that fully captures all of the domains of our conceptual model. In fact, even providing partial coverage of the proposed SEM:CY domains would require several measures. For example, there are separate measures of motivational climate for coaches, parents, and peers respectively [47–49]. These measures were designed specifically for motivational climate, so do not capture the quality of relationships. Other measures that focus on the source of influence in sport such as the *Coaching Behaviour Scale for Sport* [50] and the *Coach Athlete Relationship Scale* [51] were not designed to measure motivational climate. Using existing measures to capture youths’ sport experience would require significant time to complete which is not feasible for survey research. This is especially true for surveys that are designed to measure health and youth development across multiple domains. There are also potential gaps in existing measures of youths’ experience in sport. For example, the perceived impact of others in the sport community on motivational climate and the impact of sport on relationships with others outside sport are not captured with an existing measure.

It is also important to recognize that while some measures might initially appear to overlap with the proposed SEM:CY, there are important distinctions. The *Youth Experiences Survey- Sport* (YES-S) [52] was developed to capture positive youth development such as personal, social, and cognitive skills. As such, the YES-S [52] does not address most of the factors we propose in youths’ experience in sport. Referring back to Holt and colleagues’ [26] model, the YES-S captures the PYD outcomes box. This is further evidenced in prior research that has employed the YES-S as an outcome measure of positive youth development in sport [45, 53–55]. Therefore, the YES-S and our proposed SEM:CY measure are distinct in both purpose and in coverage.

## Methods

We will employ systematic instrument development methods to: (1) use existing literature and expert opinion to generate items that capture salient facets of youth sport experiences; (2) apply the best-available statistical techniques for item reduction to ensure the measure is brief; and (3) test the measure’s reliability and validity [56, 57]. The anticipated timeline for the study is 3 years and will be divided into three phases.

### Phase I. Identifying and testing content for the SEM:CY (year 1)

Phase I will focus on generating a pool of items that provide comprehensive coverage of the proposed domains of youth sport participation. We will review existing literature to identify existing measures that capture relevant components of the proposed domains included in our model. We will also hold focus groups and interviews with coaches, sport policy and community leaders, parents, and young athletes separately to inform item development where there are gaps not covered by existing measures. We anticipate a minimum of five focus groups with approximately five to seven participants per group to obtain saturation for each group. Focus groups will be analyzed using an inductive approach where any salient text coded and later grouped into higher order codes by at least two members of the research team. Interviews will be conducted until saturation is reached in each group. Data collection will take place concurrently with data analysis, so as to ensure that new themes are fully explored in subsequent interviews and analyses.

Once the initial pool of items is generated, we will engage with a panel of at least 15 experts in youth sport participation for initial content validation [58]. The panel will include experts with research expertise and those with significant experience in sport (e.g., coaches; leaders in sport organizations such as Sport4Life, Sport Matters, and Sport Research Intelligence Sportive). Expert panel members will be asked to provide feedback on the conceptual model of the SEM:CY measure, including the relevant constructs, their definition, and the initial pool of items for testing. At this stage, the panel will be asked to rate each item with regard to relevance, comprehensiveness, redundancy, and clarity [59]. The panel will be asked to score the items on a 5-point scale based on the degree to which the item fits with the content of the construct it is intended to measure. The panel will also be asked to provide written feedback about each item (e.g., suggested edits for wording and developmental appropriateness), the conceptual framework (e.g., identifiable gaps), and the item assessment procedure as a whole.

The content validity ratio (CVR) will be used to assess the panels’ content relevance ratings for each item and each set of items [56]. Items that are deemed relevant will be retained, items deemed relevant but in need of modification will be revised, and items deemed irrelevant will be removed. The exact number of items for the initial pool will depend on the results of the expert panel process, however we estimate the process would result in about 5 items for each domain of the conceptual model. Based on our initial conceptual model with 8 domains, this would result in an initial pool of about 40 items.

We will test items from the expert panel using cognitive interviews with approximately 10 youth in the target age range for the SEM:CY. Cognitive interviewing is a common technique used to evaluate item meaning, comprehension, and wording as well as response option choice [60–62]. We will assess children’s perceptions of difficulty and understanding of each item to ensure items are developmentally appropriate. Based on the results, items will be revised as necessary. At the end of phase I we will have an initial pool of items for testing.

### Phase II. Testing the item pool of the SEM:CY (years 2–3)

Phase II will involve testing the factor structure of the model and conducting item level analysis for item reduction and refinement. We propose to test a convenience sample of youth ages 10 to 18 (sample size estimate below) across elementary, junior, and senior high school (grades 6 to 12). The sample will be recruited from school settings, rather than from sport programs, to capture a more heterogeneous patterns of sport participation than when sampling from a particular sport club or program. Furthermore, the measure is intended for community-based youth surveys, thus a school-based sample is appropriate. Students will only be asked to complete the SEM:CY items if they have participated in organized sport in the last year.

We propose to test and refine the pool of items with 2 consecutive samples across 2 steps: (1) conduct initial item level analysis and explore the structure of the SEM:CY and (2) confirm the factor structure of the SEM:CY and conduct any further item refinement required. These steps will ensure that we will have sufficiently refined and examined the internal validity and structure of the measure prior to final validation. Moving to external validation prior to establishing internal validity can result in problems with interpretation in the meaning of external associations [63]. After the factor structure is confirmed, the relationship between the SEM:CY and perceived self-competence will be examined. Perceived competence is one of the most common psychological factors that has been examined in relation to sport participation among youth [8, 9].

### Initial item analysis and reduction

For the first sample, we anticipate requiring approximately 600 students to complete the measure. This sample will provide a ratio of 15:1 participants for each item with a 40-item scale for the exploratory factor analysis (EFA). This should provide adequate power for the EFA. While some guidelines recommend a lower 10:1 ratio, recent work suggests that these guidelines underestimate the required sample size when ideal scenarios (e.g., about 4-5 items that at least moderately load onto each factor) are not met [64, 65]. We will aim to recruit about 938 students to provide a final sample of 600. This is based on attaining a 75% response rate among students (*N* = 750) and a 75% participation rate in sports in the past year.

#### Measures and analysis

Participants will be asked to complete the pool of items for the SEM:CY, the measure will be scored with higher scores reflecting positive experiences in sport. Other information will include demographic characteristics (age, gender, and ethnicity) and questions related to involvement in sport, including breadth and intensity of participation [66] and stage of sport career [67]. This information will be important to assess the generalizability of the sample and assess whether any adjustments to the sampling procedures are required in subsequent phases (e.g., oversampling older grades to account for attrition from sport across adolescence). EFA will be used to identify the number of factors that best fit the SEM:CY and the items that measure each factor. Item level analysis will include assessing correlations among items and item-total scores. Items will deleted if: (1) they do not moderately load onto a factor, (2) they demonstrate significant cross loadings, (3) or show very weak or strong item total correlations (about .2 and .8 respectively). Prior to deleting an item, the impact on the internal consistency of the domain will be examined.

### Confirming the factor structure and initial validation

Once the revised pool of items is selected we will recruit another sample to confirm the factor structure of the measure and assess the initial validity of the SEM:CY. Finally, we will examine the time required to complete the revised SEM:CY measure to assess whether it is sufficiently brief (about 5 min) for inclusion into community-based surveys of youth [8]. This step also provides an opportunity for further item refinement if needed prior to validating the final measure. We will aim to recruit about 781 students (assuming a 75% response rate and 75% complete SEM:CY items) to provide a final sample of 500 to provide a sufficient sample to test the model in subgroups in line with recommendations for confirmatory factor analysis (CFA) [68].

#### Measures and analysis

Our demographic survey from the prior analysis and revised item pool will be administered. CFA will be used to assess whether the factor structure identified in the EFA fits in a subsequent sample. Multiple group analysis will be conducted to assess whether the structure of the SEM:CY is consistent across age and gender. The Self-Perception Profile for Children (SPPC) [69] or Self-Perception Profile for Adolescents (SPPA) [70] depending on the age of the participant will be administered to assess perceived competence. The SPPC and SPPA are valid and reliable measures of perceived competence (e.g., academic, social, and athletic) and global self-worth [69–71]. We propose a sample size of 500 to allow for analyzing the model in subgroups divided by age and gender. Multiple fit indices will be used to examine model fit [72, 73].

### Phase III. Validating the final SEM:CY measure (year 3)

Phase III will focus on a more robust test of the measures construct validity (e.g., association with youth development and well-being) and reliability (e.g., internal consistency of scales and short-term stability) and confirming any changes to the structure from phase II. We propose to recruit 391 students (assuming a 75% response rate and 75% complete SEM:CY items) to assess the final SEM:CY in 250 students ages 10 to 18. We will retest a random subset of the sample (*n* ~ 75) on the SEM:CY to examine test-retest reliability at about 2 weeks.

To test the validity of the measure we will assess its relationship with perceived competence, sport anxiety, and PYD in sport. Along with perceived competence, positive youth development in sport, and anxiety have been commonly studied in relation to sport participation. Based on both qualitative and quantitative research we would expect a positive relationship between the SEM:CY with perceived self-concept and positive youth development, and negative correlation with sport anxiety [8, 9].

#### Measures and analysis

Students will be asked to complete the final SEM:CY, the demographic survey, measures of sport participation, and SPPC/SPPA from earlier phases, along with the YES-S and Sport Anxiety Scale-2 (SAS-2) [74]. The YES-S measures positive youth development in sports across five domains: personal and social skills, initiative, cognitive skills, goal setting, and negative experiences. Prior research has found the measure to have factorial validity, convergent validity displaying positive relationships with autonomy support from coaches, self-esteem, social identity, and team cohesion [52, 55, 75, 76]. Reliability of the subscales have performed adequate to good with alphas at or above .75 [52]. The SAS-2 assesses anxiety in sport across three domains: worry, somatic symptoms, and disrupted concentration. Prior research has reported that positive relationships between the SAS-2 with mastery motivational climates and negative relationships with ego motivational climates and self-esteem. The subscales have demonstrated good reliability with alphas above .80 [74].

## Discussion

The substantial reach of sport participation among youth, coupled with the equivocal results regarding the impact of sport on health and development of youth [4, 7–9], highlight the importance of advancing research in youth sport experience. While researchers in recent years have identified some of the factors that impact youths’ sport experience [4, 27], we are still left with important gaps regarding the impact of sport on the developmental outcomes of youth.

We propose to develop the SEM:CY for on-going population health surveys and/or new surveys of child and youth health and development. The SEM:CY will provide greater quality of data on youth sport participation, by moving from simple yes/no or counts of participation, to including the salient aspects of youths’ experience. The intentional development of SEM:CY as a survey-based measure that can be embedded into existing or new surveys including population health surveys can also provide a greater quantity of data.

The improved quality and quantity of data regarding youth sport participation and experience in sport will benefit researchers from a variety of disciplines. Results from research using the SEM:CY will help identify the salient aspects of youth sport experiences that promote health and wellbeing, but also those that are harmful. These results have important implications for sport programming and practice. They can help guide programming and training efforts to avoid harms for youth, and better align sport programming with its health promoting potential. The measure can be used by researchers who wish to study youth sport participation in relation to a number of outcomes. For example, Vierimaa, Erickson, Côté, and Gilbert [77] outline a potential toolkit to measure aspects of PYD in sport. Considering the SEM:CY would include some elements identified in the toolkit (e.g., connection to coaches and teammates) which would currently require two long measures, the SEM:CY has the potential to be improve on the existing toolkit. The SEM:CY can also be useful, alongside other measures, for researchers who wish to test theories regarding how sport programing impacts youth health and development.

## Abbreviations

CFA: Confirmatory factor analysis
CVR: Content validity ratio
EFA: Exploratory factor analysis
PYD: Positive youth development
SAS-2: Sport Anxiety Scale-2
SEM:CY: Sport Experiences Measure for Children and Youth
SPPA: Self-Perception Profile for Adolescents
SPPC: Self-Perception Profile for Children
YES-S: Youth Experiences Survey- Sport
